# Fluid Intelligence and Automatic Neural Processes in Facial Expression Perception: An Event-Related Potential Study

**DOI:** 10.1371/journal.pone.0138199

**Published:** 2015-09-16

**Authors:** Tongran Liu, Tong Xiao, Xiaoyan Li, Jiannong Shi

**Affiliations:** 1 Key Laboratory of Behavioral Science, Institute of Psychology, Chinese Academy of Sciences, Beijing, 100101, China; 2 Natural Language Processing Laboratory, College of Information Science and Engineering, Northeastern University, Liaoning, 110819, China; 3 Department of Learning and Philosophy, Aalborg University, Denmark; University of Jyväskylä, FINLAND

## Abstract

The relationship between human fluid intelligence and social-emotional abilities has been a topic of considerable interest. The current study investigated whether adolescents with different intellectual levels had different automatic neural processing of facial expressions. Two groups of adolescent males were enrolled: a high IQ group and an average IQ group. Age and parental socioeconomic status were matched between the two groups. Participants counted the numbers of the central cross changes while paired facial expressions were presented bilaterally in an oddball paradigm. There were two experimental conditions: a happy condition, in which neutral expressions were standard stimuli (*p* = 0.8) and happy expressions were deviant stimuli (*p* = 0.2), and a fearful condition, in which neutral expressions were standard stimuli (*p* = 0.8) and fearful expressions were deviant stimuli (*p* = 0.2). Participants were required to concentrate on the primary task of counting the central cross changes and to ignore the expressions to ensure that facial expression processing was automatic. Event-related potentials (ERPs) were obtained during the tasks. The visual mismatch negativity (vMMN) components were analyzed to index the automatic neural processing of facial expressions. For the early vMMN (50–130 ms), the high IQ group showed more negative vMMN amplitudes than the average IQ group in the happy condition. For the late vMMN (320–450 ms), the high IQ group had greater vMMN responses than the average IQ group over frontal and occipito-temporal areas in the fearful condition, and the average IQ group evoked larger vMMN amplitudes than the high IQ group over occipito-temporal areas in the happy condition. The present study elucidated the close relationships between fluid intelligence and pre-attentive change detection on social-emotional information.

## Introduction

The nature of human intelligence is an enduring topic in scientific research of cognition. Fluid intelligence or *g* factor of intelligence has been widely adopted to describe the intelligence profile that originates from an individual’s birth, and which cannot be impacted by knowledge or experience. This factor indicates how well individuals can adapt themselves to their emotional and non-emotional environment [1]. Studies on the cognitive characteristics have consistently found that those with high IQ have better memory, attention, and cognitive control abilities than individuals with average IQ [1–2]. However, there exist different theories relating intelligence and social-emotional abilities: Spearman proposed the psychometric theory of intelligence, which posited at least modest correlations between an individual’s social-emotional abilities and his or her cognitive abilities [3]. In contrast, Gardner’s multiple intelligence theory proposed complete independence of emotional abilities and cognitive abilities or academic aptitude [4].

The investigation of the social-emotional abilities of intellectually gifted individuals dates back to Terman [5]. Several studies have provided empirical evidence of a positive relationship between intelligence and social-emotional abilities, such as ego resiliency, self-efficacy, self-esteem and reduced vulnerability [6–9]. It is observed that individual’s emotional abilities (such as, emotion perception, emotion generation, emotion understanding and emotion regulation) are correlated with fluid intelligence [10–11], and higher IQ scores are associated with faster responses during selective attention tasks involving affective information [12]. Moreover, adolescence is an extremely important period for an individual’s neurodevelopment of social-emotional abilities [13–14]. Children with higher IQ scores show better performances in emotional intelligence tests, suggesting that children with high IQ might have better emotion perception and management abilities than their average IQ peers [15]. However, it is unknown as to whether adolescents with high IQ also have better automatic neural processing of social-emotional information, and the current study is intended to investigate the relationship between fluid intelligence and neural activation of pre-attentive facial expression processes and further to provide electrophysiological proofs.

Facial expressions contain essential social-emotional information, and the electrophysiological studies using the event-related potential (ERP) technique have reported that the ERP components of P100, N170, and P300 are associated with three different stages in the perception of facial expressions [16–19]. Additionally, the automatic detection of minor changes in facial expressions is even more crucial for social-interpersonal communication [20]. To study the automatic neural processing of facial expressions, passive expression-related oddball tasks have been widely adopted [20]. An ERP component of particular interest has been visual mismatch negativity (vMMN), which is measured by subtracting the neural responses to standard, frequently presented stimuli, from those to deviant (i.e. randomly and infrequently presented) stimuli [20]. The expression-related vMMN has been regarded as an index of the automatic neural processing of facial expressions [20–30]. Moreover, Stefanics et al. [20] complemented the classic oddball paradigm with an accompanying primary task, consisting of pressing a response button rapidly in response to changes of a fixation cross, to improve the methodological validity of expression-related vMMN. They observed a significant vMMN to deviant emotional faces over the bilateral temporal-occipital electrode sites. Although the neural mechanism of the expression-related vMMN remains unclear, most researchers regarded the vMMN in response to deviant facial expressions to reflect automatic and unintentional processes in predictive memory representation [20,26,28].

The main aim of the current study was to investigate whether adolescents with different levels of intelligence have different automatic neural processing of facial expressions, and to further elucidate the relationships between fluid intelligence and the automatic processing of emotional information. In the present study, we used a similar paradigm to Stefanics et al. [20] by introducing a centrally presented visual primary task to occupy the participant’s attention, and displaying a passive emotion-related oddball paradigm on both sides of the primary task. Participants were instructed to concentrate on the central crosses and to accomplish the primary task as fast and as accurately as they could, while ignoring the bilaterally presented facial expressions. Two kinds of oddball conditions were used: a fearful oddball condition, in which fearful expressions were used as deviant stimuli and neutral faces as standard stimuli, and a happy oddball condition, in which happy expressions were used as deviant stimuli and neutral faces as standard stimuli. We compared vMMN responses for happy and fearful conditions. Based on Spearman’s psychometric theory of intelligence [3] and Zeidner et al.’s [15] performance-based emotional intelligence measurement findings, we hypothesized that adolescents with high IQ would show better automatic processing in both happy and fearful conditions than their average IQ peers, as indexed by greater vMMN amplitudes over the frontal and occipito-temporal brain areas.

## Methods and Methods

### Ethics Statement

This study was approved by the Ethics Committee of the Institute of Psychology, Chinese Academy of Sciences. Written informed consent was obtained from children and their parents.

### Participants

Two groups of adolescent males (a high IQ group and an average IQ group) were enrolled in the study. The high IQ group (n = 17, ages 13.3–14.2 years old, mean age: 13.7 years old) was recruited from a gifted education system called the “Gifted Youth Class” which offers a curriculum emphasizing the science domains, such as, mathematics, physics, chemistry, and biology. The “Gifted Youth Class” enrolls 30 children from about 1800–2000 candidates each year based on their scores on classical intelligence tests and on cognitive abilities, such as attention, memory, and executive functions. Participants in the average IQ group (n = 19, age 13.2–14.3 years old, mean age: 13.7 years old) were chosen from a conventional middle school, and had similar ages and parental socioeconomic status (SES) to those in the high IQ group. All participants were adolescent males, because most members of the “Gifted Youth Class” were boys, and selecting only adolescent male participants avoided increased variability and the need to consider additional covariates, such as girl’s pubertal status and menstrual cycle. All adolescents were right handed, with normal or corrected-to-normal visual acuity, and none had psychiatric or neurological problems.

Intelligence was measured via two classic intelligence tests: Cattell’s Culture Faire Test (55 items, 1 point/item, scale range 0–55) [31] and Raven’s Standard Progressive Matrices (60 items, 1 point/item, scale range 0–60) [32]. Participants’ IQ scores are presented in Table 1. These two instruments are regarded as the most promising tests of fluid intelligence, and have been shown to load highly on the *g* factor of intelligence [33–34]. The cutoff range of the Cattell test scores was 48–53 for the high IQ group and 36–43 for the average IQ group, whereas the Raven test range was 53–57 for the high IQ group and 42–47 for the average IQ group. A *t*-test (2-tail) analysis showed that the high IQ group achieved significantly higher intelligence scores than the average IQ group in both intelligence tests (Cattell: *t* = 5.5, *p* < 0.001; Raven: *t* = 5.1, *p* < 0.001).

**Table 1:** Participants’ means and standard deviations of IQ scores and SES characteristics.

			High IQ (n = 17)	Average IQ (n = 19)	t-test/chi-square test
IQ	Cattell test scores		51±2	39±3	*p* < 0.001
	Raven test scores		54±2	44±3	*p* < 0.001
	Standardized IQ		125±4	106±5	*p* < 0.001
SES	Maternal education	≤High school	4	5	*p* > 0.05
		Bachelor’s degree	11	11	
		Master’s/doctorate degree	2	3	
	Parental wealth	Poor	0	0	*p* > 0.05
		Lower than medium	3	2	
		Medium	10	14	
		Rich	4	3	

Since the parental SES is known to be a crucial factor in children’s cognition and emotion development [35–36], the current study also controlled SES factors, specifically parental wealth and maternal education between the two groups. Parental wealth was calculated as the family’s average income per month from their child’s birth, and maternal education was measured as the mother’s highest educational degree. A *t*-test (2-tail) comparison showed that there were no significant differences between the two groups in their SES scores (*ps* > 0.05). Detailed descriptions of the participant’s SES information are presented in Table 1.

### Stimuli and Procedure

**Fig 1:**
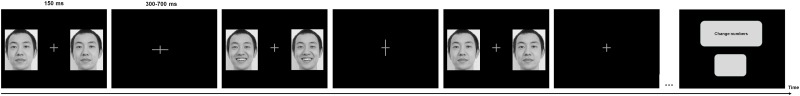
Sample stimuli and experimental procedure for one experimental block in the happy oddball condition. Two identical facial expressions were displayed bilaterally to both sides of the central fixation cross. The presentation of faces and the changes of the fixation cross were independent. The face-pair was presented on each screen for 150ms, followed by an inter-stimulus interval of 300–700 ms. The cross changed occasionally during each block, and participants were required to detect the changes and to report at the end of each block how many times the cross had changed.

Fig 1 illustrates the stimuli and procedure. The presentation screen was a computer monitor (17 inches, 1024 × 768 resolution at 100 Hz refresh rate) with a black background.

Similar to several previous vMMN studies [20,28], the primary task was displayed in the central visual field, and the expression-related oddball paradigm (facial expressions) was presented bilaterally to the central fixation cross. The expression-related oddball paradigm was displayed independently from the primary task. Participants were required to focus their attention on the primary task and to ignore the facial expression stimuli. This design guaranteed that participant’s attention was focused on the primary task and that the perception of the emotional information presented in the oddball paradigm was automatic. Participants were instructed to detect and to count how many times the central cross (“+”) changed: the horizontal line of the cross was longer than its vertical line, or the vertical line was longer than the horizontal line. The participants reported the changes at the end of each block, by key press.

Each cross change in the primary task lasted 300 ms, after which the cross returned to its original size. During each block, the cross randomly changed from zero to nine times. Participants were required to concentrate on counting the number of cross changes and to press the corresponding number stickers on the keyboard (“0” to “9”) at the end of each block to report how many times the central cross had been changed. Participants were instructed to use the left index finger to press the sticker of “0”, “1”, “2”, “3”, or “4” for the changes of zero, once, twice, three times, and four times, and to use the right index finger to press the sticker of “5”, “6”, “7”, “8”, or “9” for the changes of five times, six times, seven times, eight times, and nine times. The answer screen remained until a button was pressed. The design aimed to guarantee that the participant’s attention was fully engaged with the primary task. The accuracies and reaction time of reporting the counts were analyzed by a 2×2 ANOVA with Intelligence (high IQ, average IQ) and Expression condition (fearful, happy) as independent variables.

The facial expression images were from 10 Chinese models (5 males, 5 females) showing neutral, happy and fearful expressions. Two identical expressions from one identical model were synchronously displayed on the both sides of the central cross. Each face was displayed in light grey, with the visual angle of 6° horizontally and 8° vertically at the 65 cm viewing distance. Each face-pair was displayed for 150 ms, followed by an inter-stimulus interval of 300–700 ms. The oddball condition was either happy or fearful, with the presentation order of the two oddball conditions randomized across participants. For the happy oddball condition, happy expressions were presented as the deviant stimuli (probability of 0.2) and neutral expressions as the standard stimuli (probability of 0.8). For the fearful oddball condition, fearful expressions were displayed as the deviant stimuli (probability of 0.2) and neutral expressions as the standard stimuli (probability of 0.8). Each condition consisted of 6 practice blocks and 60 formal blocks, and there were 480 standard stimuli and 120 deviant stimuli in each expression condition. Deviants and standards were presented pseudo-randomly. There were no fewer than two standards between subsequent deviants, and no block had begun with a deviant. The vMMN responses evoked by the facial expression stimuli were analyzed to measure the individual’s automatic processing of facial expressions.

### EEG recording and analysis

The electroencephalograms (EEG) were recorded from 64 scalp electrodes via a NeuroScan Quik-Cap. The electrodes were placed according to the extended 10–20 system locations. The horizontal and vertical EOG (HEOG and VEOG) were monitored via four bipolar electrodes positioned on the outer canthi of each eye and at the inferior and superior areas of left eye, respectively. The electrode impedance was kept under 5 kΩ. The EEG signal was continuously recorded at a sample rate of 500 Hz using a nose reference, amplified using SynAmps amplifiers and online band-pass filtered at 0.05–100 Hz. The EEG signal was further epoched and averaged with 100 ms prior to and 500 ms after the stimulus onset. The pre-stimulus interval of 100 ms was used for baseline correction. Epochs were screened for artifacts: contamination by eye movements, or muscle potentials exceeding ±70 μV at any electrode were excluded from averaging. A Chi-square test showed that the remaining trial numbers were similar across groups (high IQ and average IQ) and Expression conditions (fearful condition and happy condition) (χ^2^(1,35) = 1.01, *p* = 0.3). The EEG was re-referenced to the common average potential and was filtered off-line with a zero phase shift (bandwidth: 0–30 Hz, slope: 24 dB/octave). Overall, less than 10% of the epochs were excluded from further analyses.

## Results

### Behavioral results

For the accuracies of reporting the counts, no significant main effects or interaction effects were observed. For the reaction time, the main effect of Intelligence was significant (*F*(1,34) = 4, *p* < 0.05, *η* ^*2*^ = 0.15), and post hoc pairwise comparisons (adjusted by the Sidak method) showed that the high IQ group had faster responses than the average IQ group. The Expression condition also showed a significant main effect (*F*(1,34) = 10.5, *p* < 0.005, *η* ^*2*^ = 0.24), and response speed was faster in the primary task when the happy oddball condition was presented, compared to the fearful condition (*p* < 0.05).

### ERP responses in the happy and fearful oddball conditions

**Fig 2:**
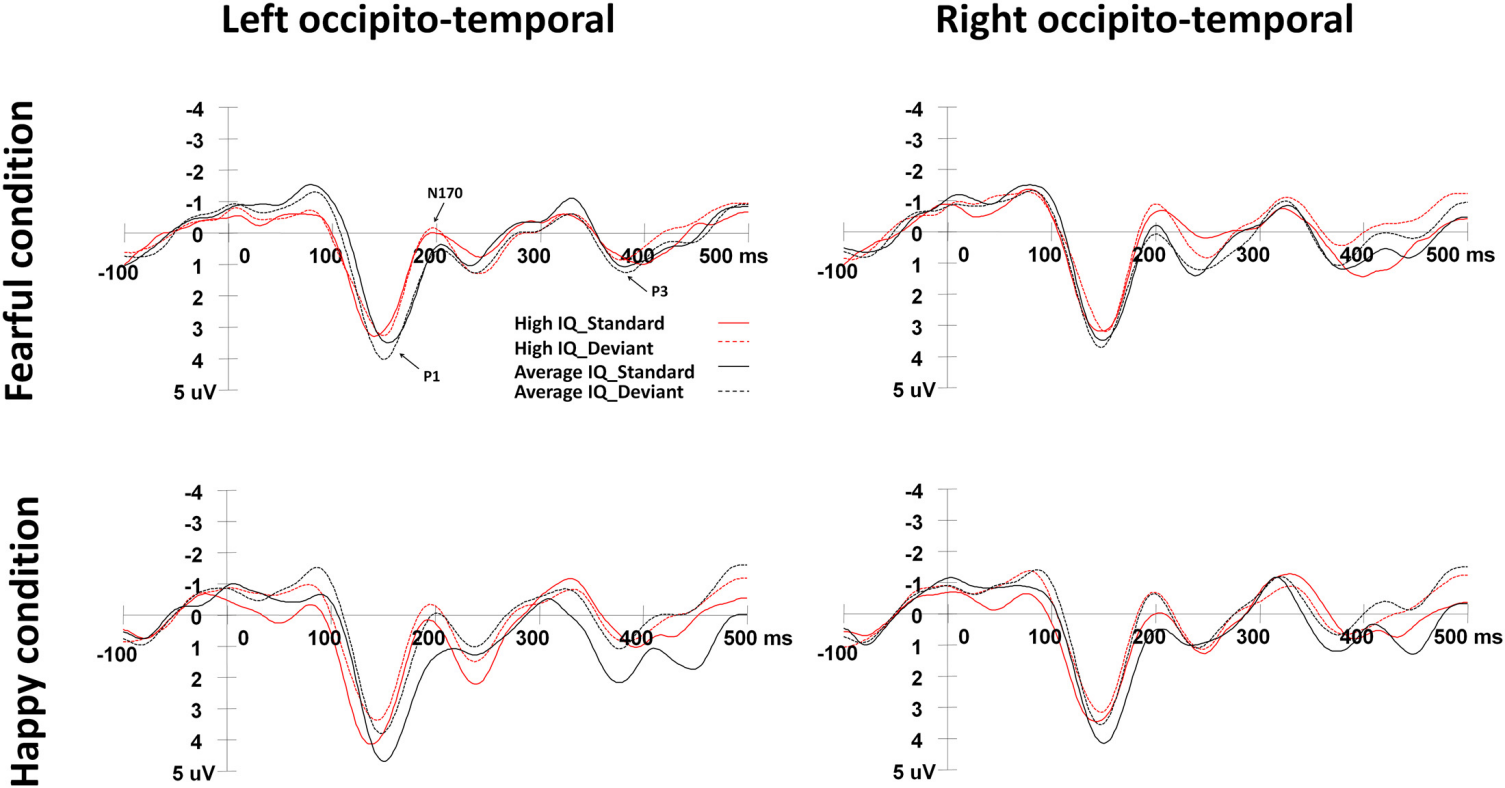
ERPs elicited by the standard and deviant stimuli in the happy and fearful oddball conditions. The left occipito-temporal waveform was the average neural activation at electrodes TP7, P7, PO7, CB1, and O1. The right occipito-temporal waveform was obtained from the average of TP8, P8, PO8, CB2, and O2.

Fig 2 shows the grand-average ERPs elicited by the standard and deviant stimuli in the happy and fearful oddball conditions. In order to analyze the automatic processing of affective deviants, vMMN was calculated by subtracting the ERP responses to standard stimuli from the ERP responses to deviant stimuli [37–40]. vMMN responses were analyzed over the frontal areas and occipito-temporal areas within three time windows: early vMMN (50–130 ms), middle vMMN (150–300 ms) and late vMMN (320–450 ms). The regions of interest (ROI) for frontal areas contained the electrodes of F1, F3, F5, F2, F4 and F6, whereas the regions of interest (ROI) for occipito-temporal areas contained the electrodes TP7, P7, PO7, CB1, O1, TP8, P8, PO8, CB2 and O2, consistent with a previous study in adults [20]. Repeated-measures ANOVAs were conducted to analyze the peak amplitudes of vMMN components, with independent variables of Intelligence (high IQ, average IQ), Expression condition (fearful, happy), and ROI (frontal, occipito-temporal).

**Fig 3:**
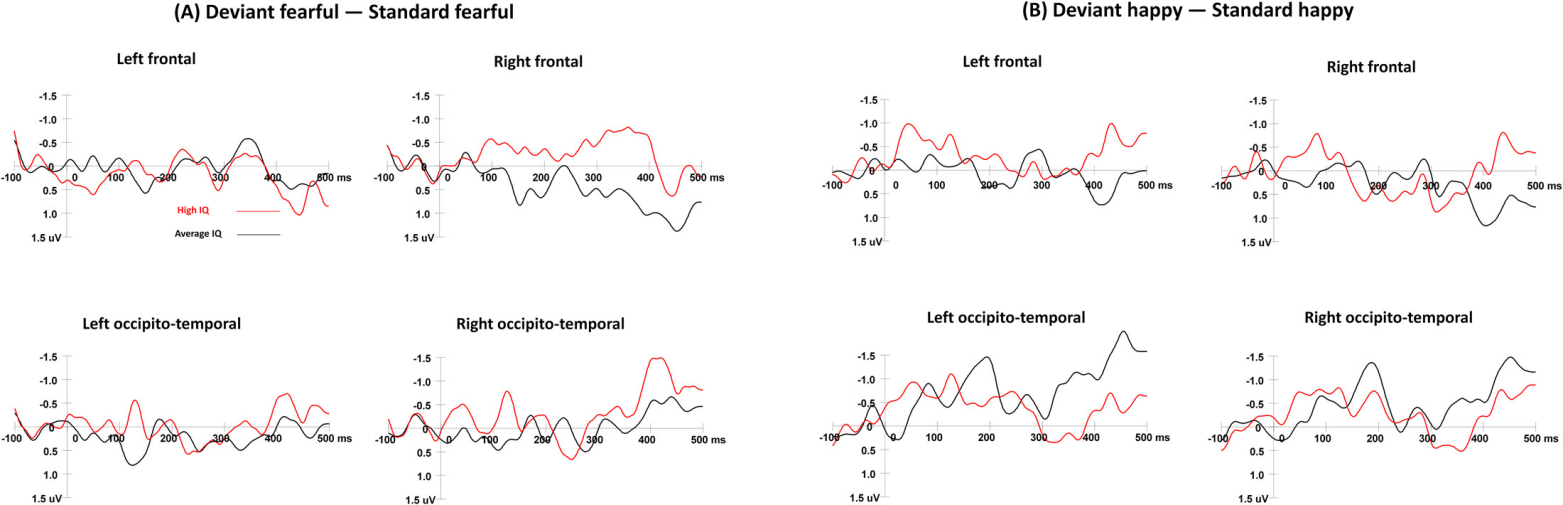
vMMN componentsin the fearful (Fig 3A) and happy oddball (Fig 3B) conditions. The vMMNs in the deviant fearful minus standard fearful condition, and the left frontal waveform was the average neural activation at electrodes of F1, F3, and F5. The right frontal waveform was obtained from F2, F4, and F6. The left occipito-temporal waveform was from TP7, P7, PO7, CB1, and O1. The right occipito-temporal waveform was from TP8, P8, PO8, CB2, and O2.

**Fig 4:**
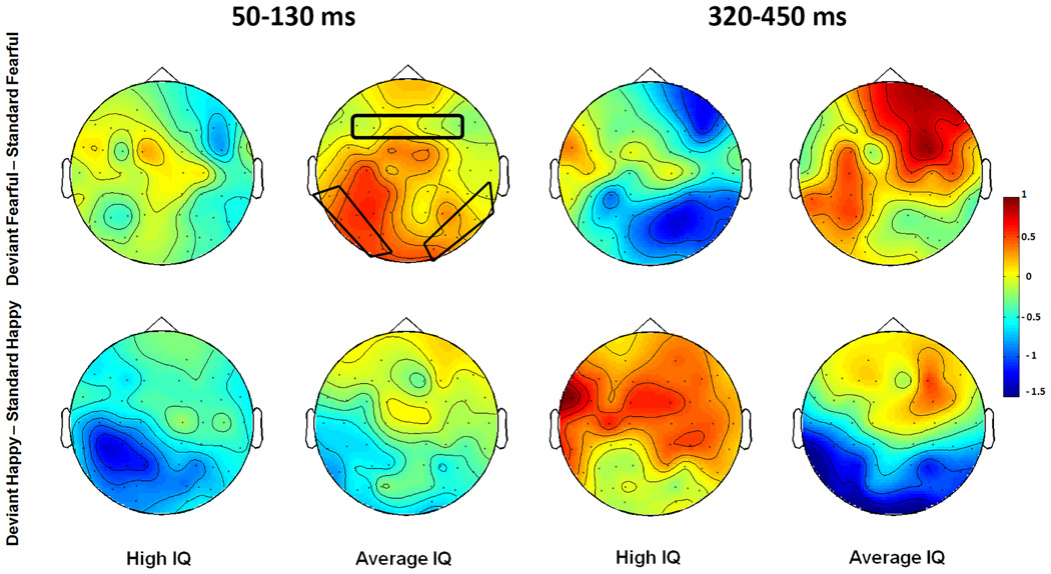
The topographic maps of vMMN components in both happy and fearful conditions during the time windows of 50–130 ms and 320–450 ms.

The means of vMMN amplitudes in both expression conditions are presented in Table 2, and the raw data of vMMN amplitudes was in the Supporting information file (S1 Data). The waveforms of vMMNs in both fearful and happy conditions are displayed in Figs 3 and 4 presents the topographic maps of Deviant-minus-Standard difference waves for two IQ groups.

**Table 2:** Means and standard deviations of vMMN amplitudes (μV) in each expression condition.

		Fearful condition		Happy condition	
Time window	IQ	Frontal	Occipito-temporal	Frontal	Occipito-temporal
50–130 ms	High IQ	-1.01±1.40	-1.24±0.90	-1.95±0.96	-1.85±0.85
	Average IQ	-1.03±1.52	-0.81±1.26	-0.95±1.05	-1.50±1.01
150–300 ms	High IQ	-1.75±2.25	-1.64±1.79	-1.33±1.27	-2.00±1.09
	Average IQ	-1.28±1.81	-1.41±1.46	-1.55±1.19	-2.26±1.95
320–450 ms	High IQ	-2.39±2.02	-2.86±1.66	-1.42±1.48	-1.47±1.53
	Average IQ	-0.83±2.06	-1.38±2.12	-0.95±1.54	-3.01±1.51

For the early vMMN (50–130 ms), the interaction of Intelligence × Expression (*F*(1,34) = 4.3, *p* < 0.05, *η* ^*2*^ = 0.11) indicated that the high IQ group had more negative vMMN amplitudes than the average IQ group in the happy oddball condition (*p* < 0.05). Post-hoc analyses showed that high IQ group had more negative vMMN responses in the happy oddball condition than in the fearful condition (*p* < 0.001).

For the middle vMMN (150–300 ms), the interaction of Expression × ROI was marginally significant (*F*(1,34) = 4.6, *p* = 0.06, *η* ^*2*^ = 0.13), whereby the occipito-temporal areas had more negative vMMN than the frontal areas in the happy condition (*p* < 0.01). There were no IQ-related main effects or interaction effects for vMMN during this epoch.

For the late vMMN (320–450 ms), the interaction of Intelligence × Expression × ROI was significant (*F*(1,34) = 8.1, *p* < 0.01, *η* ^*2*^ = 0.19), such that in the fearful condition, high IQ adolescents had more negative vMMN than average IQ adolescents over both frontal and occipito-temporal areas *(ps* < 0.05), and in the happy condition, the average IQ group had greater vMMN amplitudes than the high IQ group over the occipito-temporal areas (*p* < 0.01). Post hoc analyses also showed that over the occipito-temporal areas, high IQ adolescent had more negative vMMN in the fearful condition relative to that in the happy condition (*p* < 0.05), and average IQ adolescent had greater vMMN in the happy condition than in the fearful condition (*p* < 0.05).

## Discussion

The current study investigated the relationship between intelligence and neural activation associated with automatic facial expression processing, with the aim of adding electrophysiological evidence to the body of research. The behavioral results showed that adolescents with high IQ performed faster than their average IQ peers in reporting how many times the fixation cross changed, indicating better performance on this cognitive task [1–2]. It was also observed that participants responded more quickly in the happy condition than in the fearful condition, which might reveal that positive affect (i.e., context, or mood) facilitated their cognitive processes [41] and/or negative affect impaired the cognitive processes [42].

vMMN responses have been widely studied with a large time range from 100 ms to 580 ms over the temporal, occipital, and frontal brain areas [40], and the current vMMN responses were analyzed with different time windows of early vMMN (50–130 ms), middle vMMN (150–300 ms), and late vMMN (320–450ms). It seemed that the current expression-related, early vMMN started earlier than traditional vMMN (approximately 100 ms), which might be due to that adolescents were extremely sensitive to affective information and they showed faster pre-attentive processing on facial expressions [43–44]. The exact cognitive processes these vMMN responses reflect are still unknown, and some recent studies suggest that vMMN responses can be regarded as the perceptual prediction error signals [39–40].

The close relationships between fluid intelligence and automatic neural processes were observed for the early and late vMMNs, but for the middle vMMN. The vMMN with the epoch of 150–300 ms is regarded to reflect the difference of N170 [22]. Astikainen et al. [22] found that an ERP component at 130 ms latency was elicited in oddball but also in equal probability condition suggesting that it reflects both the detection of regularity violations (pure vMMN) and also encoding of emotional information in faces. N170 was sensitive only to emotional expressions, not stimulus probability. No significant main effect of IQ was found for the current middle vMMN, which was consistent with a prior study which reported that adolescents with different IQ levels had similar N170 amplitudes to positive and negative faces during a facial expression perception task [45], and these findings might indicate that adolescents with different IQ levels have comparable structural encoding abilities on facial expressions.

More importantly, adolescents with high IQ showed more negative amplitudes of early vMMN than adolescents with average IQ in the happy oddball condition. This suggests that adolescents with high IQ might have better pre-attentive processing of positive expressions as compared to their average IQ peers. For the late vMMN, high IQ adolescents showed greater vMMN amplitudes than average IQ adolescents in the fearful condition, and average IQ adolescents had larger vMMN over the temporal-occipital areas than high IQ adolescents in the happy condition. This demonstrates that adolescents with different intellectual levels show different perceptual bias to emotional information with different affective valences: individuals with high IQ had better automatic change detection for both happy (early vMMN) and fearful (late vMMN) minor deviants than adolescents with average IQ, whereas adolescents with average IQ might show better automatic change detection for happy deviants than high IQ adolescents during late vMMN responses. These interesting findings suggest that individuals’ fluid intelligence abilities correlate with their emotion-related behaviors [12] and social functioning [46–49].

Prior studies have showed consistently that vMMN responses correlate with individual cognitive and emotional abilities. For example, Stefanics and Czigler [20] observed that vMMN amplitudes to right hands with unexpected laterality correlated with Edinburgh handedness scores, thus revealing a close association between vMMN responses and the strength of hand-preferences. Csukly et al. [50] observed attenuated vMMN amplitudes in patients with schizophrenia. Patients’ impaired vMMN responses were significantly associated with decreased emotion recognition performance, further revealing the complex interactions between emotional and cognitive processes [51–52]. Furthermore, a relationship between vMMN responses and autism spectrum personality traits has also been demonstrated [25]. In particular, individuals with higher autism spectrum quotient scores had smaller amplitudes of vMMN responses to happy deviants. These findings illustrate that the previously discovered close associations between automatic prediction error responses (mainly for auditory MMN, aMMN) and behavioral measures in cognitive tasks also exist for vMMN responses [53–55]. Regarding the relationship between aMMN responses and cognitive abilities, a previous study adopting a classical auditory oddball paradigm with non-emotional stimuli found that highly intelligent children had better automatic detection of minor auditory changes than average IQ children. This was reflected in larger amplitudes of aMMN and late discriminative negativity (LDN) in the former [56]. Additionally, higher mental ability has been shown to be associated with larger aMMN amplitudes and shorter aMMN latencies to deviant stimuli, as compared to lower mental ability [57–60]. Furthermore, the current study also showed that adolescents with high IQ had greater vMMN to fearful deviant stimuli over the frontal areas, as compared to average IQ adolescents. This might indicate a difference in the maturity of the prefrontal cortex between the two IQ groups [51,61–65]. Generally, these findings support the view that there exist specific neural mechanisms associated with human intelligence and automatic neural processes [34,63, 66–67].

There were several limitations of the current study: first, no formal reliable measure of social cognition or emotional intelligence was used. In the future work we would measure emotional intelligence via the Mayer–Salovey–Caruso Emotional Intelligence Test [11], and consider personality traits such as depression, social anxiety, and empathy, given that these variables might modulate emotion perception at a subclinical level [68]. Second, more types of face models (e.g., simple schematic faces, complex schematic faces, and photographs of real human faces expressing emotions) might be adopted to investigate whether vMMN components are affected by lower-level physical differences among emotional stimuli.

In summary, the present study found that adolescents with high IQ can automatically perceive minor visual changes in positive expressions, as reflected in enhanced neural activation in the early vMMN. For the late vMMN, high IQ adolescents had better automatic processing of the fearful expressions than their average IQ peers, and average IQ adolescents had enhanced pre-attentive processing of happy expressions over the occipito-temporal areas. These findings demonstrated that adolescents with high IQ can process and store minor changes in both positive and negative information outside the focus of attention for further memory representation, as compared to adolescents with average IQ. The current study thus sheds light on the essential relationship between fluid intelligence and automatic facial expression perception.

## Supporting Information

S1 Data The raw data of vMMN amplitudes. S1 Data A contained the vMMN amplitudes in the 50–130 ms, S1 Data B for the vMMN amplitudes in the 150–300 ms, and S1 Data C for the vMMN amplitudes in the 320–450 ms.
(RAR)
